# Effects of sodium hyaluronate administration on levels of antioxidant enzymes, serum substance P and neuropeptide Y in patients undergoing locking plate fixation for tibial plateau fractures

**DOI:** 10.12669/pjms.39.4.7460

**Published:** 2023

**Authors:** Lei Shan, Qin Si, Peifeng Yao

**Affiliations:** 1 Lei Shan, Department of Orthopedics, Beijing Chao-yang Hospital, Capital Medical University, Beijing 100020, P.R. China; 2 Qin Si, Department of Orthopedics, Beijing Chao-yang Hospital, Capital Medical University, Beijing 100020, P.R. China; 3 Peifeng Yao, Department of Orthopedics, Beijing Chao-yang Hospital, Capital Medical University, Beijing 100020, P.R. China

**Keywords:** Locking plate fixation, Sodium hyaluronate, Tibial plateau fractures, Antioxidant enzymes, Substance-P, Neuropeptide-Y

## Abstract

**Objective:**

To analyze the effects of sodium hyaluronate administration on the serum levels of antioxidase, substance P (SP), and neuropeptide Y (NPY) in patients undergoing locking plate fixation (LPF) for tibial plateau fractures (TPF).

**Methods:**

We retrospectively analyzed the records of 66 patients with TPF who received treatment in the Beijing Chao-yang Hospital[PJMS1][2] from February 2017 to August 2020. According to the treatment records, 33 patients underwent LPF surgery (control-group), and 33 patients underwent LPF plus sodium hyaluronate treatment (observation-group). The levels of antioxidant enzymes (superoxide dismutase (SOD), catalase (CAT) and total antioxidant capacity (TAC)), SP and NPY.

**Results:**

Seven days after LPF operations, both groups showed lower levels of SOD, CAT, and TAC compared to pre-surgery levels, while levels of SP and NPY were higher. However, the observation group showed higher levels of SOD, CAT, and TAC compared to the control group, and lower levels of SP and NPY in the control group (*P*<0.05).

**Conclusion:**

The treatment of TPF with LPF plus sodium hyaluronate administration has been shown to effectively reduce oxidative stress reactions, improve SP and NPY levels.[PJMS3][4]

## INTRODUCTION

Tibial plateau fractures (TPF), fractures of the upper end of the tibia by violent impacts, are common in orthopedic practice.^1^ The tibial plateau (TP) is an essential load-carrying structure of the human knee joint. Its fractures cause the imbalance of forces between the inner and outer joint planes, and can result in damage to the cruciate ligament and meniscus.^2^ Prompt surgical treatment is required to promote the recovery of knee joint function and range by restoring the flatness of the joint surface, the integrity of the ligaments and the width of the platform.^3^ There are multiple treatments for TP, such as closed reduction, open reduction and internal fixation, and arthroplasty, but the treatment of TPF depends on the alignment of the knee joint cartilage. Locking plate fixation is one of the main employed strategies. The internal stent and ordinary steel plate used during the procedure can effectively support the affected articular surface and promote the recovery of the joint function.^4^

However; traumatic arthritis, joint swelling, joint stiffness, joint pain, and other side effects are frequent after the operation, they aggravate the degree of oxidative stress, hinder the postoperative rehabilitation, and may lead to tibial plateau deformity and a diminished joint mobility. Therefore, effective supplementary treatment plans combined with the surgical treatment are being actively sought after.^5^ Sodium hyaluronate is an important component of joint synovial fluid and of the cartilage matrix that lubricates the joint cavity. Sodium hyaluronate covers and protects the articular cartilage, it can inhibit the degeneration of the cartilage, and promotes the recovery of joint function.^6^

A few studies have investigated the combined treatment of patients with locking plate fixation and sodium hyaluronate administration for TPFs, but the effects on the levels of postoperative antioxidant enzymes and the extent of tibial plateau deformity remain unclear. In view of this, we focused on the analysis of the effects of this combined treatment on the levels of antioxidant enzymes,pain stress markers, on the tibial plateau deformity and joint ROM in 66 patients with TPF; our results favour combined usage of locking plate fixation and sodium hyaluronate for improving the prognosis of these patients.

## METHODS

We retrospectively analyzed the records of 66 patients with TPF (43 men and 23 women) who received treatment in the Orthopedic Department of Beijing Chao-yang Hospital (Capital Medical University) from February 2017 to August 2020. We divided the patients into a control-group (patients who underwent locking plate fixation, n=33) and an observation-group (patients who underwent locking plate fixation and sodium hyaluronate administration, n=33).

### Inclusion criteria:

- Patients with diagnoses of TPF confirmed by CT, X-ray and MR images consistent with the diagnostic criteria of the disease.^7^
- Patients with new, unilateral, closed fractures.
- Patients between 18 and 70 years of age.
- Patients with a first fracture and operation.
- Patients with a Schatzker classification Type-II to IV.

### Exclusion criteria:

- Patients with pathological fractures.
- Patients complicated with deep tissue infections.
- Patients with coagulation disorders.
- Patients complicated with active hemorrhage.
- Patients complicated with knee osteoarthritis.
- Patients complicated with rheumatoid arthritis.
- Patients complicated with other types of fractures.
- Patients with allergies.
- Patients with malignant tumors.

### Ethical Approval:

The ethics committee of the Beijing Chao-yang Hospital (Capital Medical University) approved this study (approval number: 2021441; July 15, 2021). Informed consent has been obtained from the patients.

### Locking plate fixation treatment:

**Fig.1:**
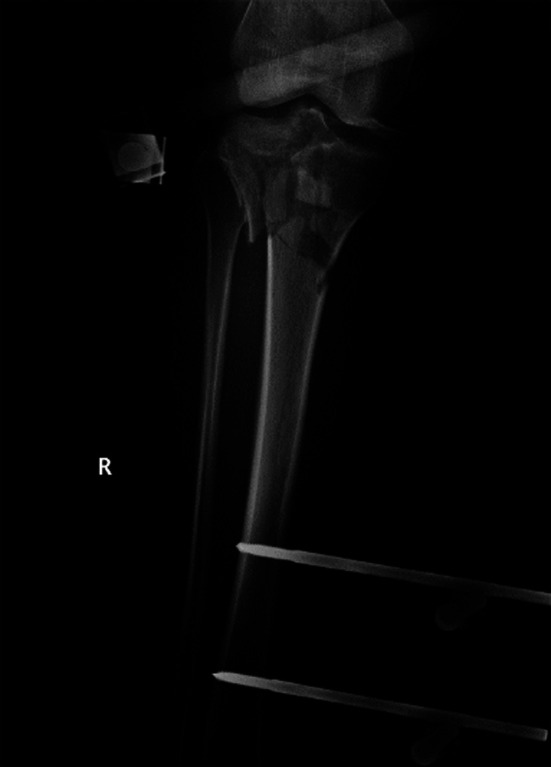
X-ray film of tibial plateau fracture before operation.

**Fig.2:**
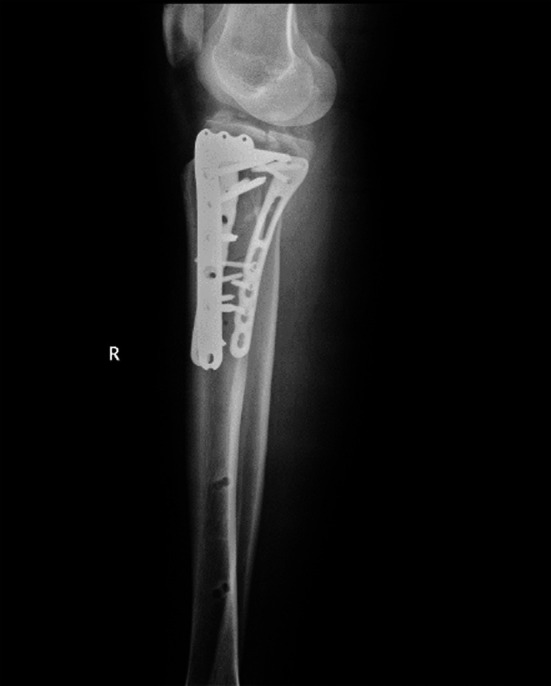
X-ray film of tibial plateau fracture after operation.

The patient was placed in a supine position and administered combined spinal epidural anesthesia. An approach was selected according to the Schatzker classification for each patient. Patients with Schatzker II and III type fractures were treated with lateral incision, and those with Schatzker IV type fractures were treated with medial incision. Locking plates were used for fixation, drainage tubes were placed routinely, and incisions were sutured layer by layer. After the operation, the affected limbs were raised, and antibiotics and analgesic prophylaxis were routinely prescribed. After the drainage tube removal, the patients were asked to join a joint rehabilitation training program (Fig.1 and Fig.2).

Locking plate fixation combined with sodium hyaluronate treatment: after closing the joint cavity, the surgeon injected 2 mL of sodium hyaluronate (Huaxi Furida Biomedical, H20133147) into the joint cavity of the patient, the injections were repeated once a week for five consecutive weeks while the joint was being drained.

### Efficacy evaluation index:

The levels of SOD, CAT, TAC, SP and NPY were measured before and seven days after the operation. The levels of SOD, CAT and TAC were measured in an automatic biochemical analyzer (Hitachi, Model 7180). The reagents were provided by Roche Biological Reagents. The levels of SP and NPY were determined using enzyme-linked immunosorbent assays (Mlbio, Shanghai, China).

### Statistical analysis:

We used SPSS v26.0 (SPSS, Chicago, IL, USA) for all statistical analyses. Categorical variables were expressed as n (%) and compared[PJMS13][14] via Fisher’s exact test or Chi-squared tests. We expressed continuous variables as means ± SD (standard deviations) and compared them via Student’s *t*-tests or Mann-Whitney’s U-tests. We considered all *p-values* <0.05 as significant.

## RESULTS

We analyzed data from 66 patients in this study. The control-group included 21 men and 12 women (age range, 24 to 81 years with an average of 48.27±12.58 years). The average time from fracture to operation was 1-6 days with an average of 3.24±1.43 days (1-6 days). The control-group included 19 Type-II, nine Type-III, and five Type-IV Schatzker classification cases; and 22 I and 11 II anesthesia ASA grades. The observation-group included 22 men and 11 women (average age, 49.45±12.21 years, ranging from 26 to 82 years). Their average time from fracture to operation was 1-6 days with an average of 3.09±1.33 days (1-6 days). The observation-group included 18 type-II, eight Type-III, and seven Type-IV Schatzker classification cases; 20 patients anesthesia ASA Grades-I and 13 patients with Grade-II. We found no significant differences in the general data between the two groups (*P*>0.05; Table-I).

**Table-I:** Comparison of general information between the two groups.

	n	Gender (male)	Age (years)	Time from fracture to operation(d)	Schatzker typing (n)			ASA rating (n)		LPF	

					II	III	IV	I	II	Single LPF	Double LPF
Control-group	33	21(63.64%)	48.27±12.58	3.24±1.43	19	9	5	22	11	15	18
Observation-group	33	22 (66.67%)	49.45±12.21	3.09±1.33	18	8	7	20	13	13	20
*χ^2^*/*t*	-	0.067	-0.387	0.444	0.419			0.262		0.248	
*p*-Value	-	0.796	0.700	0.658	0.811			0.609		0.804	

The levels of SOD, CAT and TAC between the two groups before operation were similar (*P*>0.05), but the indexes of antioxidant enzymes in the two groups were significantly lower seven days after the operation. We found the levels of SOD, CAT and TAC in the observation-group were significantly higher than those in the control-group after the operation (*P*<0.05; Table-II). Before the operation, the levels of SP and NPY between the two groups were similar (*P*>0.05). Seven days after the operation, the pain stress markers in the two groups were significantly higher than before the operation, and the observation-group had significantly lower levels than the control-group (*P*<0.05; Table-III).

**Table-II:** Comparison of antioxidant levels between the two groups (*χ̅*±*S*, U/mL).

	n	SOD		CAT		TAC	

		Day before surgery	7 days after operation	1 day before surgery	7 days after operation	1 day before surgery	7 days after operation
Control-group	33	74.41±12.04	63.42±10.25^^a^^	41.96±6.94	33.38±6.15^^a^^	11.89±1.73	7.68±1.65^^a^^
Observation-group	33	74.13±13.34	68.30±9.45^^a^^	43.14±5.92	40.12±4.60^^a^^	12.03±1.75	10.56±1.59^^a^^
t	-	0.091	-2.010	-0.738	-3.543	-0.325	-7.187
p-Value	-	0.928	0.049	0.463	0.001	0.747	<0.001

******Note:******

a compared with the admission day in this group *P<*0.05.

**Table-III:** Comparison of the pain stress level between the two groups (*χ̅*±*S*, U/ml).

Group	n	SP(μg/ml)		NPY(pg/ml)	

		Day before surgery	7th day after operation	Day before surgery	7th day after operation
Control-group	33	3.67±0.83	7.16±0.89^a^	152.88±15.18	193.51±18.16^a^
Observation-group	33	3.75±0.73	4.83±0.88^a^	154.94±16.47	166.39±17.28^a^
t	-	-0.453	10.691	-0.528	6.213
p-Value	-	0.652	<0.001	0.599	<0.001

******Note:******

a compared with the admission day of this group *P<*0.05.

## DISCUSSION

The results of this study showed that LPF plus sodium hyaluronate treatments can reduce oxidative stress reactions, improve SP and NPY levels. Wang ZH et al treated patients with unstable femoral trochanteric fracture by locking plate fixation.^8^ The results showed the Harris hip joint scores increasing after the operation, and the fracture reduction rate reached 71.79%, indicating that locking plate fixation can reduce the fractures, shorten the time for fracture healing and full weight bearing, and promote the recovery of the hip joint function. Yu TJ et al^9^ treated Sanders II and III closed calcaneal fractures with locking plates, and they found that the Böhler and Gissane joint angles increased after the operation, and that the AOFAS and FADI scores also increased significantly.

However, the recovery period after locking plate fixations is relatively long. In addition, under the influence of disease, trauma, surgical stress, and other factors, the ligaments and synovium in the joint are prone to congestion and swelling, and the tissue edema accompanied by joint pain and stiffness with other symptoms can lead to traumatic arthritis. The prognosis of which is difficult to assess. Therefore, relieving joint pain while operating can further improve joint function by fully protecting the articular cartilage.^8^

Sodium hyaluronate can reduce the permeability of the body’s synovium, and it reduces the amount of fluid accumulated in joints. Akyildiz S et al found through animal experiments that sodium hyaluronate can increase the amount of ossification in rats with tibial fractures.^10^ Our results showed that seven days after the operations, the levels of SOD, CAT and TAC seven days after the operations were lower than those before the operation in the two patient groups, but the comparison between groups showed that the levels in the observation-group were higher than those in the control-group (*P*<0.05), suggesting that addition of sodium hyaluronate treatments to the surgical approach in the treatment of TPFs alleviates oxidative stress reactions. SOD, CAT, and TAC are common indicators of oxidative stress in the clinic, and research has shown that oxidative stress damages microvessels on the damaged joint surface and affects the quality of bone healing.^11^ On the basis of LPF, the use of sodium hyaluronate treatments inhibits macrophage tissue chemotaxis: the combination of the hyaluronate with glycoproteins forms a polymer that prevents their participation in inflammatory reactions, alleviating the inflammatory process and promoting damaged cartilage repair while reducing the oxidative stress.^5^interleukin-1 receptor antagonist protein (IRAP SP and NPY are common indicators of pain stress. SP and glutamate can coexist in neural terminals, where they have a role in pain signal transmission; NPY is widely distributed in the central and peripheral nervous systems and has been implicated in pain pathways.^12^,^13^

In addition, the levels of SP and NPY in the two groups were higher seven days after the operation than before operation, but the levels in the observation-group were lower than those in the control-group, suggesting that the treatment with sodium hyaluronate reduced SP and NPY levels. The results were similar to those of Stitik TP et al. and Khan IU et al.^14^,^15^ During the LPF operations, the content of sodium hyaluronate in the joint cavity gets significantly reduced due to the repeated need for wound washing. The damaged synovium cannot effectively replenish the lost sodium hyaluronate fast enough to maintain cartilage protection and lubrication. Therefore, the intense friction of damaged joint surfaces during rehabilitation training can lead to wear particles and pain receptor stimulation causing the pain stress to increase abnormally.^14^

Sodium hyaluronate injected into the joint after LPF can lubricate the joint, cushion the vibration and create a protective barrier on the synovium and cartilage surfaces to prevent the destruction of the cartilage matrix until the synovium can secrete enough sodium hyaluronate by itself.^16^ Yang X et al found in their meta-analysis that arthroscopy combined with intra-articular sodium hyaluronate injections for the treatment of knee osteoarthritis can improve the knee joint function of patients, alleviate their symptoms, and improve their quality of life.^17^ We believe that our approach allowed the patients to carry out rehabilitation training in a timely manner to improve their prognosis by ensuring the best rehabilitation effects and improve the deformity of the tibial plateau and the range of motion of the joints.^16^ Therefore, in this present study, LPF combined with sodium hyaluronate administration was found to be effective for the management of patients with TPF.

### Limitations of the study:

The study only included records of patients with TPF admitted by the orthopedic department of our hospital, the cohort was small, few indicators were surveyed, and the follow-up observation times were short. Hence a large sample size and multicenter study is needed to confirm our findings.

## CONCLUSION

The treatment of TPF with LPF plus sodium hyaluronate administration has been shown to effectively reduce oxidative stress reactions, improve SP and NPY levels, thereby improving the patient’s prognosis.

### Authors’ contributions:

**LS:** Conceived and designed the study.

**QS** and **PY:** Collected the data and performed the analysis.

**LS:** Was involved in the writing of the manuscript and is responsible for the integrity of the study.

All authors have read and approved the final manuscript.
